# Attentive Listening and Confirmation Key Aspects in Health Promotion Encounters With Patients in Telephone Counselling

**DOI:** 10.1111/scs.70141

**Published:** 2025-10-24

**Authors:** Margaretha Larsson, Catharina Gillsjö, Anna Sundberg, Maria Zakrisson, Irene Eriksson

**Affiliations:** ^1^ School of Health Sciences University of Skövde Skövde Sweden; ^2^ College of Nursing University of Rhode Island Kingston Rhode Island USA; ^3^ Närhälsan, Södra Ryd Skövde Sweden; ^4^ Home Health Care in Skövde Municipality Skövde Sweden; ^5^ Department of Health Sciences University West Trollhättan Sweden

**Keywords:** communication, health promotion, phenomenology, telenursing, telephone counselling

## Abstract

**Introduction:**

Health‐promoting telephone counselling is a growing but underexplored area of nursing, where district nurses (telenurses) play a key role in supporting patients' health and autonomy without physical presence. While this form of care is widespread, time constraints, emotional strain, and system limitations may challenge telenurses' ability to create meaningful, person‐centred encounters. Gaining insight into how telenurses experience health‐promoting interactions is essential for improving care quality and support systems. This study contributes new knowledge by exploring the lived experiences of telenurses in such encounters, offering perspectives that can inform education, policy, and practice development.

**Aim:**

The study aim was to describe health‐promoting encounters with patients in telephone counselling as experienced by telenurses.

**Methods:**

A qualitative, phenomenological interview study, grounded in a Reflective Lifeworld Research approach, was conducted with 12 participants in western Sweden.

**Findings:**

The findings comprise one key essence with three constituents. The essence of the phenomenon of health‐promoting encounters with patients in telephone counselling as experienced by telenurses involves a moment of confirmation and the creation of a sense of security for the patient. The phenomenon is further described with all its variations and nuances through its three constituents: creation of mutual understanding and meaningfulness, attentiveness to patients in their context, and a calm conversation without stress.

**Conclusion:**

The results in this study reveal that telenurses have an intention to create mutual understanding and a sense of security to achieve meaningfulness in the encounter with patients. Confirming the patient through attentive listening and being present in the encounter facilitates a holistic perspective based on the patient as a whole, which is in accordance with a person‐centred approach. The results in this study contribute to knowledge in the area of health‐promoting encounters with patients in telephone counselling, a topic with limited research.

## 1 Introduction

Health‐promoting care aspires to support and preserve human health in a process that enables humans to take control over and improve their health [1]. Health‐promotion nursing actions, as described by Berg and Sarvimäki [2], act as a way to encounter the needs of persons and groups in their efforts to manage the health challenges they meet in daily life. An essential starting point is the dialogue between persons and nurses, which can enable nurses to use their knowledge to facilitate the development of the person and to empower them to improve his or her health. Nurses can, through active listening and by considering each person's narrative, along with reviewing examination results, use a person‐centred care approach, which forms the basis of establishing a relationship of mutual respect and equality [3]. Burdett and Inman [4] state that a person‐centred care approach can be combined with a health promotion approach as a sufficient way to support a person's health. Furthermore, by listening to the person's narrative, their internal and external resources, expectations, and barriers to these can be made visible. In working within health promotion, the WHO [1] highlights that a person's health literacy level is an important aspect to consider and support. Health literacy concerns people's ability to acquire, understand, and apply health‐related information. A person's level of health literacy matters when they make judgements and decisions related to healthcare, healthcare prevention, and health promotion, and is important for maintaining and improving a good life [5].

Providing care by telephone is practiced internationally [6], and telephone care services are often available during office hours in primary healthcare and 24 h a day and 7 days a week, for example, by such services as Health Direct Australia and Healthcare Direct Sweden. The purpose of telephone care services is to offer accessible and secure high‐quality care as well as to deliver care efficiently [7]. Telephone care services are often provided by registered nurses. In Sweden, just over 50% of primary healthcare services are provided by the public service and the rest are private [8]. Together they employ approximately 17,000 nurses [9], who manage just over 4 million calls per year [8]. The registered nurses often also hold a specialist nurse qualification, with the most common being a district nurse specialist. Similar to nursing standards in several countries globally, specialist nurses in Sweden are certified and have a postgraduate level of higher education, including a master's degree. An integrative review revealed that quality in nurses' telephone encounters with persons/callers is associated with factors such as having completed a nurse specialist education programme, their experience, competence, and communication skills, employing person‐centredness, as well as the available healthcare resources and their organisation and working conditions [10]. In Sweden, the competence description for district nurses prescribes a health‐promoting approach and the ability to provide support for people of all ages and medical conditions [11], and, when working with the telephone service, assessing acute and temporary health problems, they provide support and advice, and sometimes refer callers to another care service [12]. Nurses working within the telephone healthcare service are hereafter called telenurses.

Telenurses are described as being professionals who work in very remote settings [13], and who work with a computer and wear a headset while managing a flow of unsorted incoming calls [14]. Often, they are supported by a computerised decision support system (CDSS). In Sweden, the CDSS contains medical quality assured decision‐making databases, structured by symptoms, and lists the symptoms and their related causes. These are categorised by the CDSS algorithm into five grades of emergency. Telenurses can search for symptoms such as dizziness or anxiety, for example, and obtain support on how to ask further questions, as well as links to information about patient associations and websites to focus on, for example, specific advice about children. The main focus of the CDSS is on somatic symptoms of illness, and the medical content in the CDSS is continually updated by a medical expert group [15]. The CDSS has been described by telenurses as being supportive as well as inhibiting, particularly when the assessment made by the CDSS does not align with their own professional opinion [16, 17]. Telenurses need to have professional knowledge and competence, particularly when considering that the CDSS does not allow the linking of several symptoms together, which can hinder nurses' ability to capture a holistic picture of the caller's situation [18]. Providing care by telephone has been described as demanding a high level of knowledge in both nursing and in communication skills [19]. An integrative review reveals that factors such as a nurse's level of education and competence have an impact on the quality of care provided in telenursing [10]. A systematic review describes, from a patient perspective, that trust in nurses depends on the nurses' knowledge and level of commitment in the dialogue to create a relationship and address contextual issues [20].

To work within a health promotion approach, nurses can be hindered by organisational cultures [21], such as when their manager states that they are taking too much time [22]. A challenge telenurses have to manage is decision‐making under time pressure as they have a time‐limited amount of time to dedicate to each call. This can vary between 6 and 10 min, which might cause stress and difficulties when aiming to create health‐promoting person‐centred encounters. Research describes that stressed nurses, when working within telephone healthcare, are more likely to be affected by impaired concentration, attention and memory, and also with making more conservative decisions and recommendations [23]. Becoming emotionally concerned is central to the telenurse's experiences of difficult calls. Difficult calls are accompanied by feelings such as inadequacy, uncertainty and anxiety, which can be described as emotional tension. Emotional tension refers to situations when the caller's expressed emotions were conveyed to the telenurses and this altered their state of mind [24]. Health promotion by telephone, described from a nursing perspective, aims to support and strengthen the person's own resources in resolving their health issues [25]. Research exploring telenurses' perspectives describes that an essential strategy they adopt is to be calm and to feel secure in themselves, especially when they manage calls that they experience as being difficult [26]. While most research focuses on conditions or difficulties related to telenursing, very few studies have explored health‐promoting encounters with persons in a telephone situation from the telenurse's perspective and even fewer have adopted a phenomenological perspective. This study will add to the limited research in the area of health‐promoting encounters in telephone counselling from the perspective of telenurses. This research is essential as telenurses are at the frontline of decision‐making and emotional care in a setting where non‐verbal cues are absent, time pressure is high, and organisational constraints are significant. Understanding their perspective is central to improving practice, education, and informing policy development. Therefore, this study aims to describe health‐promoting encounters with patients in telephone counselling as experienced by telenurses.

## 2 Methods

### 2.1 Design

This qualitative study followed a Reflective Lifeworld Research (RLR) approach [27, 28], grounded in a Reflective Lifeworld Research approach in phenomenology and the lifeworld perspective, to gain a deepened understanding of the phenomenon of health‐promoting patient encounters in telephone counselling as experienced by telenurses through the description of their lived experiences. In using the RLR approach, an open and reflective approach with the bridling of one's pre‐understandings is required to acquire a multifaceted description and understanding of the phenomenon [27, 28].

### 2.2 Ethical Considerations

All steps in this study followed national ethical regulations and conform to the ethical principles of the Declaration of Helsinki [29]. According to Swedish legislation, Swedish Ethical Review Authority approval was not needed for this study, as it did not include sensitive personal data, nor was there a risk of affecting the participants' health and well‐being [30]. The four ethical principles of respect for autonomy, beneficence, non‐maleficence, and justice were taken into consideration throughout the project. The managers of six primary health care centres approved telenurses' participation in the study and forwarded the request and information to district nurses who met the inclusion criteria. All participants were informed about the study and the confidential nature of their participation and about their rights as participants, including their right to withdraw without explanation. The participants provided oral and written informed consent before participating. Data are presented at group level.

### 2.3 Setting and Participants

The participants in this research study comprised district nurses working in primary healthcare centres in the regional healthcare service or the private sector in the western region of Sweden. The inclusion criteria for participation were being an educated district nurse, able to speak Swedish, and having experience in telephone counselling. A convenience sampling method was used. Contact was made with managers in 21 primary healthcare centres through e‐mail, and six managers agreed to forward the information. In total, 12 district nurses participated: 11 women and one man, aged between 34 and 62 years. The length of experience as a registered nurse varied from 10 to 41 years, and as a district nurse, from 1 to 30 years. The length of experience as a telenurse in telephone counselling varied from 1 to 18 years.

### 2.4 Data Collection

The data collection was performed in Swedish through semi‐structured interviews (Supplementary file 1) [31] conducted over three weeks in February–March 2023, using a Reflective Lifeworld Research (RLR) approach according to the principles suggested by Dahlberg et al. [27] and Dahlberg [32]. The authors continuously reminded each other of the importance of keeping an open mind and of the bridling of any pre‐understandings and acted accordingly in the data collection phase. The development of the semi‐structured interview guide was grounded in the theoretical underpinnings of the RLR approach, the life world perspective, and person‐centred care literature. The interview guide consisted of open questions to encourage reflection and dialogue. It was reviewed by experienced qualitative researchers in the research team and piloted with one participant. Based on this pilot, one adjustment was made with the addition of a question that also became the opening question. This change was made to support the participants' orientation towards the studied phenomenon and to encourage reflection and openness in their responses.

The majority of the interviews were carried out by the third and fourth author through face‐to‐face interviews at each participant's workplace. One interview was conducted via videolink. The interviews started with the question “What does health promotion work involve for you?” to start the dialogue around the subject before moving on to ask questions such as “What is your experience of a health‐promoting encounter in telephone counselling?”; “What made the encounter health‐promoting?”; and “Would you please describe an encounter that was not health‐promoting?” Follow‐up questions such as “Can you please describe further?”; “How did it make you feel?”; and “What were your thoughts?” were used to deepen the dialogue and reflection regarding the subject matter. Each interview ended with the question “Is there anything else you would like to talk about in regard to health‐promoting encounters with patients in telephone counselling” to further deepen the reflection. This ending question generated additional descriptions of the studied phenomenon. The interviews lasted between 13 and 28 min and were audio‐recorded and transcribed verbatim.

### 2.5 Data Analysis

The analysis was carried out according to the principles of RLR proposed by Dahlberg et al. [27] and Dahlberg [28] and entailed placing a focus on meanings that constitute and describe the phenomenon. Initially, the second, third, and fourth authors listened through all recorded interviews to obtain a holistic understanding of the meaning of the phenomenon. Secondly, the transcribed text was read several times with an open‐minded approach and reflection and a conscious bridling of any pre‐understandings of the phenomenon. This approach was adopted throughout the entire analysis process. The third and fourth authors continued the analysis by identifying and marking meaning units, that is to say, smaller segments or words that described the phenomenon. In order to allow for a new understanding of the phenomenon, reflection and discussion took place to bridle any pre‐understandings. In order to understand the meaning units and search for latent content in the text, questions were asked to increase the understanding and determine whether they meant anything other than the explicit. The meaning units were grouped into 14 clusters based on similarities and differences and named based on their meaning and characteristics. To discern and understand the phenomenon, the process involved an iterative movement throughout the analysis in which clusters, as parts, were viewed in light of the text as a whole. By reflecting on the meaning of the clusters and whether similar responses meant the same or vice versa, more similarities and differences were discovered, and the clusters were condensed into three clusters in dialogue with the second author. Thereafter, all authors were involved in the analysis, and the findings were discussed and reflected upon. Reflection on the meaning of the clusters, seen as being part of the whole, simplified the discernment of the essence of the phenomenon. Together, the essence and the three constituents—creation of mutual understanding and meaningfulness, attentiveness to patients in their context, and a calm conversation without stress—formed a new whole, producing a new understanding of the phenomenon, which is presented and illustrated by quotations translated from Swedish to English.

## 3 Findings

The findings comprise one key essence and three constituents. The essence of the phenomenon of health‐promoting encounters with patients in telephone counselling as experienced by telenurses involves a moment of confirmation and the creation of a sense of security for the patient. Central to the encounter is to understand the person and their life situation by being an attentive listener. This also entails offering time and knowledge by being present in the moment during the patient encounter, and to equally decide what promotes the patient's well‐being and health. The phenomenon is further described with all its variations and nuances through its three constituents: creation of mutual understanding and meaningfulness, attentiveness to patients in their context, and a calm conversation without stress.

### 3.1 Creation of Mutual Understanding and Meaningfulness

The telenurses emphasise that mutual understanding and meaningfulness are key to health‐promoting telephone counselling. They act as fellow human beings, confirming and respecting patients, especially in cases of loneliness or mental illness. By making encounters person‐centred and equal, they aim to create security and meaning. Achieving mutual understanding, regardless of the encounter's content, fosters a sense of meaning for the patient. One telenurse describes this as “health can be promoted in many different ways, I mean that a nice treatment on our part can perhaps promote the patient's health in the patient's disease state& a warm and pleasant treatment, then I still think that we have promoted health in that difficult moment”. (IP12).

The telenurses create meaningful, health‐promoting encounters by offering self‐care and lifestyle advice, empowering patients to take control of their health. They encourage patient participation to reduce the need for visits with a nurse or physician. While patients may expect immediate bookings or prescriptions, mutual understanding is achieved when they grasp the reasons behind the self‐care advice. A telenurse describes how “you feel that you have a rather large role there as a nurse or a telenurse. Based on what we are talking about. You don't always have to book a visit here”. (IP9).

The telenurses find that what is meaningful varies for each patient. Informing, educating, and motivating patients to make lifestyle changes can lead to “aha moments,” helping them realize what they can improve. These encounters often serve as a “wake‐up call,” sparking interest in bettering their health. When patients understand that they can influence their situation through effort, the encounter becomes health‐promoting. One telenurse describes: “so I don't expect this person to get well and be able to turn their life around just because they talked to me for ten minutes, I don't think so, but I think that you plant a seed and this person might also have had a seed sown.” (IP10).

The telenurses experience that they need to make sure that the patient has understood the health‐promoting self‐care advice and that it takes place in consultation and in mutual understanding. They describe that the patient's attitude determines the possibility of creating health‐promoting encounters. It helps if the patient is interested, receptive to information and health‐promoting advice, and is willing to do their own work in contributing to a change. The path to motivating the patient is described by a telenurse: “Walk on a new path, it takes time& it is difficult before it becomes a habit with whatever it is that you have to change. Many times they call and want help and then they are already on their way”. (IP4).

Telenurses' ability to achieve mutual understanding varies based on both their experience and that of the patients. They find it challenging to give advice if they are unsure whether the patient understands or accepts it. Factors affecting health‐promoting encounters include the patient's gender and age, with mutual understanding often perceived as being easier to achieve with women and middle‐aged patients. Personal chemistry also plays a role, as some patients prefer speaking with specific telenurses. As one telenurse describes, “my experience says that women are a little more receptive than men and a little more interested in jumping on that train. There are many who actually, well, you can do it yourself”. (IP5).

When discussing self‐care or lifestyle changes, some patients may react with anger, aggression, or threats. If the encounter is not health‐promoting, telenurses may request a return call due to time constraints or the patient's unreceptiveness. It is crucial not to become defensive or break guidelines in these situations. The telenurses find it helpful to end the call and reconnect later when the patient is calmer and more open to mutual understanding. One telenurse describes how “it is very clear that then they are not satisfied and it does not feel good that they have not achieved anything, but I also cannot go against what and how they do things at healthcare centres in which directions they take things”. (IP7).

The telenurses experience mutual understanding and meaning when patients are satisfied with the counselling. Positive emotions such as joy, pride, and accomplishment strengthen their ability to provide health‐promoting care. While negative feelings can drain energy, these diminish as telenurses gain experience with health‐promoting approaches in telephone counselling.

### 3.2 Attentiveness to Patients in Their Context

Health‐promoting patient encounters during telephone counselling are experienced by telenurses as patient encounters where they listen in and see the unique person in context. A good health history is described as being a basis that contributes to being able to view the person behind the patient. The health history is considered to be most complete when it covers all parts in, and about, the patient's life, both in terms of their background and current state of health, and based on their physical, psychological, social, and existential context. A telenurse describes that the encounter entails:

to listen in to where this person is … what kind of experiences they have in the past, but that you are attentive, that you listen to what the person has to say. (IP6).

The telenurses experience that attentiveness is essential to understanding the patient's life and needs. They use open and closed questions to gather a complete picture, forming the basis for health‐promoting encounters. These encounters include discussing the patient's lifestyle—diet, exercise, weight, substance use, and mental well‐being. One telenurse says that “Lifestyle is extremely important. If you get a grasp of how the patient lives, you can also be better at supporting with health‐promoting thinking”. (IP3).

Listening to a patient's expectations, hopes, and fears helps create a health‐promoting encounter. The telenurses describe how common reasons for contact include colds, lifestyle issues, and mental illness. When patients change their reason for calling, it is crucial to listen carefully and “read between the lines” to understand their situation. Although it is a challenge to not see the patient physically, with experience, the telenurses become better at perceiving unspoken needs. One telenurse describes how “The basis of our care is to be attentive to the patient's needs, which makes me able to hear this little cry between the lines”. (IP11).

The telenurses consider that most encounters by phone are health‐promoting in some sense, as patients express that they usually have been helped and are satisfied. However, some patients become angry, dissatisfied, and irritated and question the telenurse's opinions, which makes it challenging for the telenurses to focus and remain attentive to the patient's overall situation in their context in the effort to create a health‐promoting patient encounter. Situations that may trigger patient dissatisfaction include unmet expectations for a physician's appointment or prescription, discrepancies between patients' perceived urgency and the telenurse's assessment, system‐related constraints such as limited availability, or communication challenges when patients feel unheard, particularly in emotionally charged circumstances.

### 3.3 A Calm Conversation Without Stress

The telenurses describe that, to create a calm, health‐promoting encounter, they must be responsive, show interest, let patients speak without interruption, and maintain a respectful, positive tone. Asking questions, confirming, and summarising help acknowledge the patient's perspective, fostering a supportive conversation. The telenurses highlight time as a key challenge in delivering health‐promoting telephone counselling. They note that facilitating a calm, stress‐free conversation requires time, but with only about four or 5 min permitted for listening, there is often limited opportunity for confirmation and creating a sense of security, which is essential for a health‐promoting encounter. One telenurse describes how “it's hard when you have seven minutes for each call, you know it's calling, you see it's calling, now the next one is calling, it's very stressful” (IP1). Having sufficient time for engaging in a stress‐free conversation is felt to be important to obtain a complete health history, make an assessment, and provide advice in a health‐promoting manner. The importance of talking respectfully and with a happy and humble tone despite a lack of time in this situation is highlighted.

The telenurses describe challenges in creating a health‐promoting patient encounter when they are new to the profession due to difficulties in mastering aspects of telephone counselling, such as maintaining structure and focus. They find it helpful to start by addressing the patient's expectations, hopes, fears, and resources. Additional challenges include balancing efficiency with multitasking to focus on the patient and making decisions with a holistic view of the patient's overall situation.

The telenurses use different strategies, both consciously and unconsciously. In some cases, the patient's question is allowed to dominate, to create a calm and stress‐free conversation that is health‐promoting during telephone counselling. While some of these strategies are used in challenging situations, they also represent approaches that telenurses employ more generally to support health‐promoting encounters. One strategy is to ensure that the assessment and advice are accurate and safe for the patient. Another strategy is to rely on their own experience, education, competence, or ideas that arose from their own perceptions and experiences of care. Other strategies are to consult with colleagues or, alternatively, allow a colleague take over the call, using motivational calls (MI) and using the CDSS. One telenurse describes: “Then maybe it's better to offer someone else to talk to, maybe a colleague who gets to take that conversation next time, because usually you have a barrier like this so it's hard to break it”. (IP8).

The telenurses develop new strategies for facilitating calm, health‐promoting conversations by collaborating and reflecting as a team. They emphasise following medical guidelines for structure and patient safety, and feel more secure by following up with care plans. Health‐promoting encounters require preparation and concentration to reduce stress. When the telenurses are well‐prepared, focused, familiar with their tools, and in a good mood, conversations are calm and effective, even under time constraints.

## 4 Discussion

The aim of this study was to describe health‐promoting encounters with patients in telephone counselling as experienced by telenurses with a qualitative Reflective Lifeworld Research approach. The essence of the phenomenon of health‐promoting patient encounters in telephone counselling as experienced by telenurses involves a moment of confirmation and the creation of a sense of security for the patient. Central to this encounter is understanding the person and their life situation, enrolling the patient's health history as a basis that contributes to being able to view the person behind the patient. Early research describes how patients' narratives enable nurses to be more attentive to the person [33]. In line with the lifeworld approach, the essence identified in this study—confirmation and the creation of a sense of security—captures what is most fundamental across the variations of health‐promoting encounters in telephone counselling. While strategies such as active listening, being calm, and offering time illustrate *how* telenurses work in practice, these strategies converge towards the deeper meaning of enabling patients to feel confirmed as persons and secure in their situation. Hence, the essence should not be understood as one single aspect among others but as the underlying structure that gives coherence to the different ways in which health promotion is realised in telephone counselling. In the current study, the telenurses use strategies such as active listening, being calm, and offering time to support patients to reveal details about their expectations and concerns related to the contact they have with the telenurses. Strategies for the creation of a calm conversation were to be responsive, showing interest, and letting the patient finish talking, as well as adopting a humble tone, asking questions, confirming, and summarising. Earlier research has described that an essential strategy for telenurses is to be calm and secure in themselves to manage calls [26], and the importance of establishing a positive opening of the conversation to create a relationship with the patient [34]. The strategies used by the telenurses in the current study are described as being important to allow them to work in accordance with a person‐centred care approach [3, 33]. It seems to be crucial that the telenurses explore what expectations the patient has for the call in order to be able to facilitate a person‐centred and health‐promoting approach [4]. Hence, the telenurses need to use active listening and consider the patients' narratives with the intention of identifying their expectations as well as their internal and external resources [3, 35]. A previous study shows that it is important for telenurses who practise person‐centred care by telephone to remould their role, which means moving between being the expert and being a partner, listening carefully and allowing for an equal level of communication to respect both the patients' and the telenurses' knowledge and expertise [36].

In this study, the results suggest that offering time and knowledge by being present in the moment during the patient encounter and, equally, deciding what promotes the patient's well‐being and health is one other aspect that is important to the phenomenon of health‐promoting patient encounters in telephone counselling. In the present study, the telenurses describe that the patients sometimes expect to be given an appointment with a physician or to receive a prescription, not to be provided with self‐care advice. Furthermore, this demands that the telenurses adopt a person‐centred approach in which the telenurses listen to the patient's health history to really understand the person and thus be able to motivate the patient when they are expecting something else. Bostrom et al. [36] argue that traditional ways of providing information are rarely satisfying to either nurses themselves or to the patients. This means that nurses may not be taking the patient's own resources and knowledge into account and instead adopt an approach where they present traditional information and advice, which are described as medical “truths” by Boström et al. [36]. Therefore, the telenurses must be attentive to their own attitudes, their own role, and their contributions to the communication by telephone. The patient's own expectations could be related to norms such as gender, life situation, and culture. It is important for the telenurses to have an awareness of these, because such norms can affect the patient's expectations of health care, but they must also be aware that there is an inevitable imbalance of power between patient and nurse [37]. For example, the patient may have stereotypical perceptions of the physicians' or nurses' role in the health care system. Such values need to be challenged, i.e., nurses need to have a norm‐critical approach, which requires self‐reflection and needs to be made clear and included in nursing education programmes [38]. How telenurses meet expectations in the health promotion work is something that needs to be managed daily and which places demands on the telenurses' ability to motivate patients when they actually expect something else from their phone call.

The creation of mutual understanding and meaningfulness is described in the present study as being central to establishing a health‐promoting encounter on the telephone. The telenurses act as fellow human beings, confirm, and are respectful of the patient as a human in a situation, to achieve mutual understanding. An integrative review from the telenurses' perspective reveals that mutual understanding is created when they confirm the patient's narrative and reach an agreement around assessments and interventions to manage the situation [34]. Rørtveit et al. [20] describe, from a patient perspective, that experiences of trust relate to the nurses' approach. This study's results describe that telenurses strive to make the encounters person‐centred and equal, and that, when working in this way, an important aspect is to consider the patient's health literacy level. This relates to how persons navigate complex health systems and their ability to access, understand, appraise, and apply information [39]. A patient's health literacy level relates to factors such as age, place of residence, and level of education [40]. From the telenurses' perspective, this illustrates the importance of them having enough knowledge and skills to make correct assessments and provide adequate advice, particularly as a lack of knowledge and experience can be a hindrance for providing good and safe care [41].

## 5 Strengths and Limitations

The choice of method to conduct the study was a strength, as RLR, proposed by Dahlberg et al. [27] is a well‐known and appropriate approach in regard to the studied phenomenon. In line with RLR, the inclusion criteria were inclusive in regard to age, gender, ethnicity, work experience, and geography to achieve diversity in experience and rich data, which strengthened the trustworthiness (credibility, dependability, conformability, transferability) of the study [27]. The concept of trustworthiness is four‐fold and considered to entail the concepts of credibility, dependability, conformability, and transferability [42]. The limited number of male participants can be seen as a limitation. However, it can be seen to be in line with the existing reality in the role, where there is a predominance of female telenurses in Sweden. The interviews were preceded by a pilot interview to ensure that the participant had the experience requested in the study and was able to answer the questions needed to inform the aim of the study. The pilot interview resulted in a change to the questions asked. The opening question was changed in the subsequent interviews to improve the participants' openness, reflection, and orientation towards the phenomenon in order to collect rich data on their experiences and the meaning of the phenomenon. This change strengthened the credibility of the study [27, 42]. Furthermore, the open question asking whether the participant had anything to add with regard to the phenomenon deepened the reflection, and further descriptions were gained which also strengthened the credibility of the study. The duration of the interviews ranged from 13 to 28 min, and potential concerns may be raised regarding whether the duration of the interviews might have influenced the credibility of the study. However, the character of the questions, being designed to encourage reflection and to provide rich descriptions, as well as the use of follow‐up questions and the final question at the end of the interview, further strengthened the credibility and dependability of the study. The final question may be viewed as being repetitious, but it may also be viewed as an iterative question, and it did work as a probe to further extend and deepen the reflection. The pilot interview and the subsequent interviews were conducted by two of the researchers, which strengthened the dependability in relation to stability in data collection. The authors' preunderstandings were bridled by keeping an open mind and by engaging in constant reflection throughout the study to enrich its trustworthiness in regard to credibility, dependability, and conformability [27, 42]. The transparency and description of the process, considerations, and choices made in carrying out the study strengthen the credibility and increase the possibility for transferability to similar contexts.

## 6 Conclusions

The results of this study reveal that telenurses strive to create mutual understanding and a sense of security in order to achieve meaningfulness in their encounters with patients. By confirming the patient through attentive listening and presence, they foster a holistic perspective grounded in the patient's unique life context—an approach aligned with person‐centred care. This study contributes to theoretical knowledge by deepening the understanding of how health promotion is enacted in practice within the constraints of telephone counselling, where physical absence and time pressures challenge relational care. It offers practical insights into the core elements that support health‐promoting encounters—such as attentiveness, emotional presence, and space for dialogue—which can inform telenursing education, reflective practice, and organisational support structures. In a field with limited prior research, these findings highlight the importance of enabling conditions that allow for relational continuity and presence, even in remote and time‐constrained care settings.

## Author Contributions

Conception and design of the study: M.L., C.G., A.S., M.Z. and I.E.; data collection: A.S. and M.Z.; data analysis and interpretation of data: M.L., C.G., A.S., M.Z. and I.E.; drafting or revising the article: M.L., C.G. and I.E.; final approval of the version to be submitted: M.L., C.G., A.S., M.Z. and I.E.

## Ethics Statement

As stated in the manuscript, ethics approval was not needed for the study according to Swedish legislation (30). However, the study complies with national ethics regulations and standards for research and conforms to the Declaration of Helsinki (29). The participants gave oral and written informed consent before participating. Data are presented at the group level.

## Conflicts of Interest

The authors declare no conflicts of interest.

## Data Availability

The data that support the findings of this study are available on request from the corresponding author. The data are not publicly available due to privacy or ethical restrictions.
